# Assessment of the vulnerability of alpine grasslands on the Qinghai-Tibetan Plateau

**DOI:** 10.7717/peerj.8513

**Published:** 2020-02-06

**Authors:** Meng Li, Xianzhou Zhang, Yongtao He, Ben Niu, Jianshuang Wu

**Affiliations:** 1 Lhasa National Ecological Research Station, Key Laboratory of Ecosystem Network Observation and Modelling, Institute of Geographic Sciences and Natural Resources Research, Chinese Academy of Sciences, Beijing, China; 2 University of Chinese Academy of Science, Beijing, China; 3 College of Resources and Environment, University of Chinese Academy of Sciences, Beijing, China; 4 Institute of Environment and Sustainable Development in Agriculture, Chinese Academy of Agricultural Sciences, Beijing, China

**Keywords:** Alpine grasslands, Exposure, Sensitivity, Resilience, Vulnerability

## Abstract

Assessing ecosystem vulnerability to climate change is critical for sustainable and adaptive ecosystem management. Alpine grasslands on the Qinghai-Tibetan Plateau are considered to be vulnerable to climate change, yet the ecosystem tends to maintain stability by increasing resilience and decreasing sensitivity. To date, the spatial pattern of grassland vulnerability to climate change and the mechanisms that vegetation applies to mitigate the impacts of climate change on grasslands by altering relevant ecosystem characteristics, especially sensitivity and resilience, remain unknown. In this study, we first assessed the spatial pattern of grassland vulnerability to climate change by integrating exposure, sensitivity, and resilience simultaneously, and then identified its driving forces. The results show that grasslands with high vulnerability were mainly located on the edges of the plateau, whereas alpine grasslands in the hinterlands of the plateau showed a low vulnerability. This spatial pattern of alpine grassland vulnerability was controlled by climatic exposure, and grassland sensitivity and resilience to climate change might also exacerbate or alleviate the degree of vulnerability. Climate change had variable impacts on different grassland types. Desert steppes were more vulnerable to climate change than alpine meadows and alpine steppes because of the high variability in environmental factors and their low ability to recover from perturbations. Our findings also confirm that grazing intensity, a quantitative index of the most important human disturbance on alpine grasslands in this plateau, was significantly correlated with ecosystem vulnerability. Moderate grazing intensity was of benefit for increasing grassland resilience and then subsequently reducing grassland vulnerability. Thus, this study suggests that future assessments of ecosystem vulnerability should not ignore anthropogenic disturbances, which might benefit environmental protection and sustainable management of grasslands on the Qinghai-Tibetan Plateau.

## Introduction

Assessment of the vulnerability of ecosystems which focuses on the assessment of the potential effects of perturbations on a specific ecosystem has become a major topic in the field of global change ecology and sustainability research (Urruty, Tailliez-Lefebvre & Huyghe, 2016; Xu et al., 2016). It is well recognized that climate change is driving structural and functional changes in natural ecosystems (Tilman & Lehman, 2001; Venter et al., 2016), especially in the sensitive and vulnerable alpine and montane ecosystems (Crossman, Bryan & Summers, 2012). In the future, increasing climate variability and more frequent and intense extreme events are likely to increase risks to natural ecosystems (Kharin et al., 2013; Li et al., 2018). Some studies have confirmed that some species or ecosystems have a high ability to adapt to climate change, whereas others are suffering negative consequences (Kozlov, 2008; Neilson et al., 2005). Therefore, identification and prioritization of the vulnerable areas are crucial to mitigate the threat of climate change to ecosystems and to achieve sustainable management and adaptive conservation of natural ecosystems.

Assessment of vulnerability aims to measure the ability of an ecosystem to resist and cope with environmental perturbations (Crossman, Bryan & Summers, 2012). A more effective and accurate vulnerability assessment framework was presented by Intergovernmental Panel on Climate Change (IPCC), which described the vulnerability as a function of ecosystem sensitivity and adaptivity to climate change with a different character, magnitude, and rate (IPCC, 2001). This definition integrates the multiple properties and processes of ecosystems, including sensitivity, resilience, and exposure, into vulnerability assessment (Xu et al., 2016). Sensitivity measures the ability of ecosystems to withstand environmental disturbances, and is quantified by the magnitude of vegetation response at the moment of the climate anomaly (De Keersmaecker et al., 2015). Resilience refers to the ability of ecosystems to recover to its original state after the disturbance, or the magnitude of absorbed disturbance by the ecosystem before the ecosystem’s structure begins to change (Holling, 1996; Scheffer et al., 2001). Exposure refers to the degree of climate disturbance experienced by a species or ecosystem and represents the rate of migration that species need to follow climate change (Loarie et al., 2009).

More recently, various methods have been developed to quantify the vulnerability of ecosystems, including the comprehensive index method (Nguyen et al., 2016), the quantitative evaluation model method (Zhao & Wu, 2014), and the scenario analysis method (Woznicki et al., 2016). However, some shortcomings remain in those methods. For example, although many studies have used the comprehensive index method to assess the vulnerability, the indicator selection and weight determination of the indices remain controversial. The quantitative evaluation model method is limited by the complexity of the dynamic vegetation. Although scenario-based assessment provides an advance in predicting future ecological vulnerability, it requires large quantities of historical data to drive the model (Jiang et al., 2018). As a consequence, the assessment of vulnerability is labor-intensive and may be less accurate. With the development of remote sensing, some studies have quantified the components of vulnerability, such as sensitivity and resilience, based on the short-term dynamics of vegetation and climatic factors at regional and global scales (De Keersmaecker et al., 2015; Li et al., 2018; Li et al., 2019a), which provides a feasible and efficient method to assess ecosystem vulnerability.

Alpine grasslands dominate the Qinghai-Tibetan Plateau, covering 1.28 × 10^8^ hm^2^ and accounting for more than 65% of its total land area (Long et al., 1999). These grasslands are critical for livestock husbandry and environmental security (Yang et al., 2017; Yao et al., 2012). As a result of the harsh characteristics of high altitude, drought, and cold, alpine grasslands on the Qinghai-Tibetan Plateau are sensitive and vulnerable to climate change and human activities (Harris, 2010; Shang et al., 2014). In the past decades, most grasslands have experienced accelerated warming (Chen et al., 2013; Li et al., 2010). Moreover, the effects of human disturbances (mainly referring to livestock grazing) on alpine grasslands have also intensified. Currently, under the influences of overgrazing and climate change, nearly 40% of the grassland has been degraded, resulting in decreases in plant diversity and productivity of alpine grasslands (Zhang et al., 2015b). Grassland degradation and even desertification are likely to increase ecosystems vulnerability and seriously threaten ecological security and regional sustainable development. Assessment of the vulnerability of alpine grasslands on the Qinghai-Tibetan Plateau is a necessary first step to explore target measures to eliminate or alleviate negative influences. Although several studies have explored the effects of climate change on grassland ecosystems (Fu, Shen & Zhang, 2018; Shen et al., 2015), to our knowledge, no studies have illustrated the patterns of the vulnerability of alpine grasslands on the Qinghai-Tibetan Plateau based on the IPCC definition of vulnerability.

In this study, we first integrated sensitivity, resilience, and exposure to quantify the vulnerability of alpine grasslands on the Qinghai-Tibetan Plateau according to the IPCC framework. We then compared the differences in vulnerability among different eco-geographical regions, grassland types, and grazing intensities. We aimed to (1) identify where grasslands on the Qinghai-Tibetan Plateau are most vulnerable, and (2) detect what aspects determine their vulnerability. Overall, we hope this study can assist policymakers and stakeholders in achieving sustainable and adaptive management of alpine grasslands in the context of climate change on the Qinghai-Tibetan Plateau.

## Materials and Methods

### Study area

**Figure 1:**
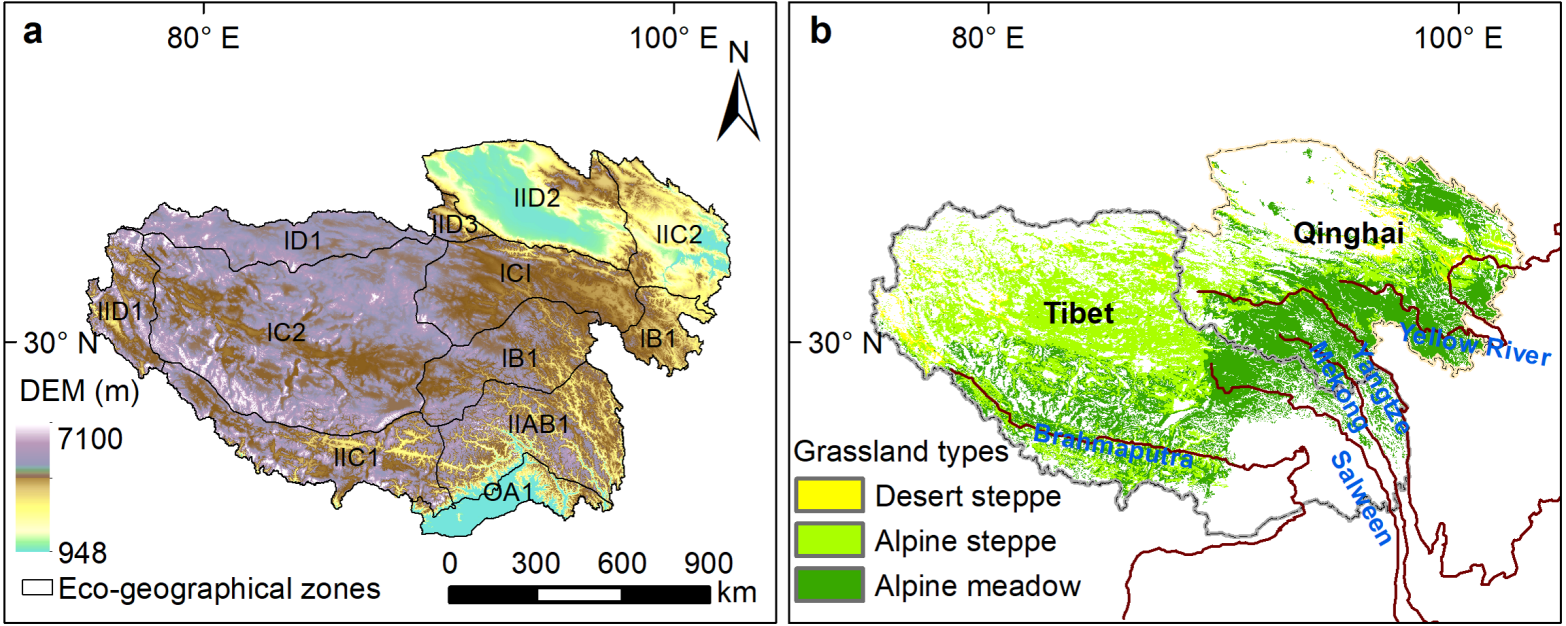
Eco-geographical regions (A) and alpine grassland types (B) on the Qinghai-Tibetan Plateau. IB1, Golog-Nagqu high-cold shrub-meadow zone; ICI, Southern Qinghai high-cold meadow steppe zone; IC2, Qiangtang high-cold steppe zone; ID1, Kunlun high-cold desert zone; IIAB1, Western Sichuan-eastern Tibet montane coniferous forest zone; IIC2, Eastern Qinghai-Qilian montane steppe zone; IIC1, Southern Tibet montane shrub-steppe zone; IID2, Qaidam montane desert zone; OA1, Southern slopes of Himalaya montane evergreen broad-leaved forest zone; IID1, Nagri montane desert-steppe and desert zone; IID3, Northern slopes of Kunlun montane desert zone.

The Qinghai-Tibetan Plateau, known as “the water tower of Asia”, is vital to safeguard ecological security for both China and South Asia (Yao et al., 2012). The plateau is located in the arid alpine climate zone with an average elevation over 4,000 m above sea level. The annual average temperatures range from −15 °C to 10 °C (You et al., 2013). The precipitation distribution in the Qinghai-Tibetan Plateau shows much spatial heterogeneity, with mean annual precipitation being about 50–150 mm in the northwest and 300–450 mm in the southeast. From southeast to northwest, grassland types alter from humid alpine meadow, semi-arid alpine steppe, to arid alpine desert-steppe (Fig. 1A). The Qinghai-Tibetan Plateau is separated by the Tanggula Mountains into two regions, Qinghai province and the Tibet Autonomous Region (Fig. 1B). The northern Qinghai-Tibetan Plateau, also named “Changtang”, contains the largest national nature reserve in China. The Three Rivers Headwater region contains high biodiversity and a fragile and sensitive ecological environment, and is the birthplace of China’s three major rivers, the Yangtze River, the Yellow River and the Lancang River (Shao et al., 2017).

### Data collection

The normalized difference vegetation index (NDVI) has been widely applied in regional ecosystem monitoring and evaluation. In this study, we downloaded the Version 6 NDVI from the monthly MOD13A3 product of the Moderate Resolution Imaging Spectroradiometer (MODIS), which is at 1 km spatial resolution (https://lpdaac.usgs.gov/get_data/data_pool). The monthly NDVI data were developed using the maximum value composition method, and have been calibrated for geometric effects, atmospheric effects, and cloud contamination. The NDVI dataset was smoothed and reconstructed with the Savitzky–Golay method to exclude the effects of cloud, snow, and ice contamination (Chen et al., 2004).

Monthly mean temperature and total precipitation data between 2000 and 2017 were collected from the National Meteorological Information Center (NMIC) of the China Meteorological Administration (CMA) (http://www.cma.gov.cn/). The origin meteorological data were interpolated into raster surfaces at the same spatial resolution as the NDVI data using ANUSPLIN 4.3 (Hutchinson, 2004). ANUSPLIN is a professional interpolation software in which one or more influence factors can be included as covariates to improve the interpolation accuracy, especially for time series of meteorological data (Hutchinson, 2004). Our previous studies have shown the accuracy of interpolation data (Chen et al., 2014; Li et al., 2019b).

Information on the distribution of grasslands was obtained from the vegetation atlas of China with a scale of 1:1,000,000 (Chinese Academy of Sciences, 2001). Eco-geographical zones adopt the framework scheme of the China eco-geographic regional system drawn up by Zheng (1996).

### The vulnerability of the grassland ecosystem

In this study, we quantified the alpine grassland ecosystem vulnerability to climate change at the regional scale by simultaneously considering sensitivity, resilience, and exposure (Li et al., 2018). The formula is as follows:

(1) \begin{eqnarray*}& & \mathrm{V I}=((\mathrm{EI}\times \mathrm{SI})/(1+\mathrm{RI}))^{1/2}\end{eqnarray*}

where VI is the vulnerability index, SI is the sensitivity index, RI is the resilience index, and EI is the exposure index.

In recent years, a novel method was presented by De Keersmaecker et al. (2015) to simultaneously quantify vegetation sensitivity and resilience under short-time climate anomalies, which has been successfully used at the regional and global scale (Geng et al., 2019; Seddon et al., 2016). In this study, we accepted this empirical methodology to quantify the sensitivity and resilience of alpine grasslands productivity to short-term climate variability for each pixel on the Qinghai-Tibetan Plateau. This method assumes that the change of vegetation index is a linear combination of climatic factors and the changes in vegetation index in the early stage. We took the grassland NDVI anomaly as the dependent variable and the temperature, precipitation (or water availability), incoming radiation anomaly and NDVI anomaly history as independent variables to model the vegetation response to short-term climate anomalies, as the following equation (autoregressive model, AR1):

(2) \begin{eqnarray*}& & {\mathrm{NDV I}}_{\mathrm{t}}=\mathrm{\alpha }\times {\mathrm{T}}_{\mathrm{t}}+\mathrm{\beta }\times {\mathrm{P}}_{\mathrm{t}}+\mathrm{\gamma }\times {\mathrm{R}}_{\mathrm{t}}+\mathrm{\delta }\times {\mathrm{NDV I}}_{\mathrm{t}-1}+\varepsilon \end{eqnarray*}

where NDVI_t_ is the standardized NDVI anomaly at time t, T_t_ is the standardized temperature anomaly at time t, P_t_ is the standardized precipitation index at time t, R_t_ is the standardized radiation index at time t, NDVI_t−1_ is the standardized NDVI anomaly at time t−1 and ε is the residual error. α, β, γ, and δ are model coefficients. To ensure the comparability among the three coefficients, all-time series of NDVI and the three climate variables were de-trended and then transformed to *z*-score anomalies using variables’ means and standard deviations.

The sensitivity index was quantified with the combination of α, β, and γ. The coefficient α is the temperature sensitivity metric denoting the response of grassland NDVI to instantaneous variation in temperature, where a higher α value indicates a higher sensitivity of grasslands to temperature, and vice versa. Similarly, β and γ represent drought sensitivity metric and radiation sensitivity metric, respectively. To calculate the sensitivity metric, we first quantified the relative contributions of temperature, precipitation, and radiation to the grassland sensitivity index for each pixel based on a principal component regression (PCR). Second, the sensitivity scores for each climatic variable were calculated by the ratio of NDVI variance and each climate variance in time series. Then, the ratios were weighted using the contribution of each climatic variable to NDVI variation. Finally, the sensitivity index was calculated by summing the sensitivity scores. More detailed methods and the R script for calculating the sensitivity index can be found in Seddon et al. (2016).

The coefficient δ is an indication of the similarity between the current state and the previous state. If δ is large, ecosystem anomalies at time t are strongly dependent on the anomaly at time t−1, and the ecosystem recovers slowly from any disturbance. Conversely, ecosystems with smaller δ values tend to recover quickly from any disturbance. As such, δ can be considered as a resilience metric, with higher absolute δ values representing lower resilience (De Keersmaecker et al., 2015; Li et al., 2018). Thus, in this study, the resilience index is equal to 1−δ.

The exposure index was defined as the ratio of the temporal climate gradient to the spatial climate gradient (Loarie et al., 2009). The formula is as follows:

(3) \begin{eqnarray*}& & {\mathrm{E}}_{\mathrm{tem}}=(\textdegree \mathrm{C}\times {\mathrm{year}}^{-1})/(\textdegree \mathrm{C}\times {\mathrm{km}}^{-1})\end{eqnarray*}

(4) \begin{eqnarray*}& & {\mathrm{E}}_{\mathrm{pre}}=(\mathrm{mm}\times {\mathrm{year}}^{-1})/(\mathrm{mm}\times {\mathrm{km}}^{-1})\end{eqnarray*}

(5) \begin{eqnarray*}& & {\mathrm{E}}_{\mathrm{rad}}=(\mathrm{MJ}\times {\mathrm{year}}^{-1})/(\mathrm{MJ}\times {\mathrm{km}}^{-1})\end{eqnarray*}

(6) \begin{eqnarray*}& & \mathrm{EI}=\mathrm{\alpha }\times {\mathrm{E}}_{\mathrm{tem}}+\mathrm{\beta }\times {\mathrm{E}}_{\mathrm{pre}}+\mathrm{\gamma }\times {\mathrm{E}}_{\mathrm{rad}}\end{eqnarray*}

where α, β, γ, and δ are the coefficients from Eq. (2). The temporal trend from 2000 to 2017 was calculated using least squares regression, and the spatial gradient was calculated using the average maximum technique based on a 3 × 3 grid cell neighborhood (Burrows et al., 2011).

Sensitivity, resilience, exposure, and vulnerability of grassland ecosystems were normalized between 0 and 100 using the minimum and maximum values. To measure the relative vulnerability and ensure its spatial comparison, we binned grassland areas into five levels of ecosystem sensitivity, resilience, exposure, and vulnerability, such that each bin had an equal interval, and labeled these bins as slight (0–20), low (20–40), moderate (40–60), high (60–80), and extreme (80–100).

### Grazing intensity

The main human activity on the Qinghai-Tibetan Plateau is livestock grazing, the extent of which was calculated based on the statistical data with the following equation:

(7) \begin{eqnarray*}& & \mathrm{GI}=\mathrm{S/ A}\end{eqnarray*}

where GI represents the index of grazing intensity (GI), S denotes the numbers of livestock in each county, and A represents the area of available natural grassland in each county (ha).

The numbers of livestock were acquired from the yearly “Statistical Yearbook of Tibet” and “Agricultural Statistics Manual of Qinghai Province” from 2000 to 2017. Sheep and large livestock were included in the original livestock data. In this study, different animals were standardized into sheep units (SHU) with the criterion that one sheep is equal to one SHU and one large livestock is equal to four SHU (Fan et al., 2010). The average grazing intensity from 2000 to 2017 for 78 counties (16 counties in Qinghai province and 62 counties in the Tibet autonomous region) was used to explore the effects of human activities on the vulnerability index as well as the three components, including sensitivity, resilience, and exposure.

### Statistical analysis

We employed the analysis of variance (ANOVA) to analyze the difference of grassland vulnerability and its components among different eco-geographical regions and also among different vegetation types. General linear models (GLMs) were conducted to explore the relative effect strengths of grassland types and grazing intensity to the spatial patterns of grassland vulnerability and its components.

## Results

### Spatial patterns of grassland vulnerability and its components

**Figure 2:**
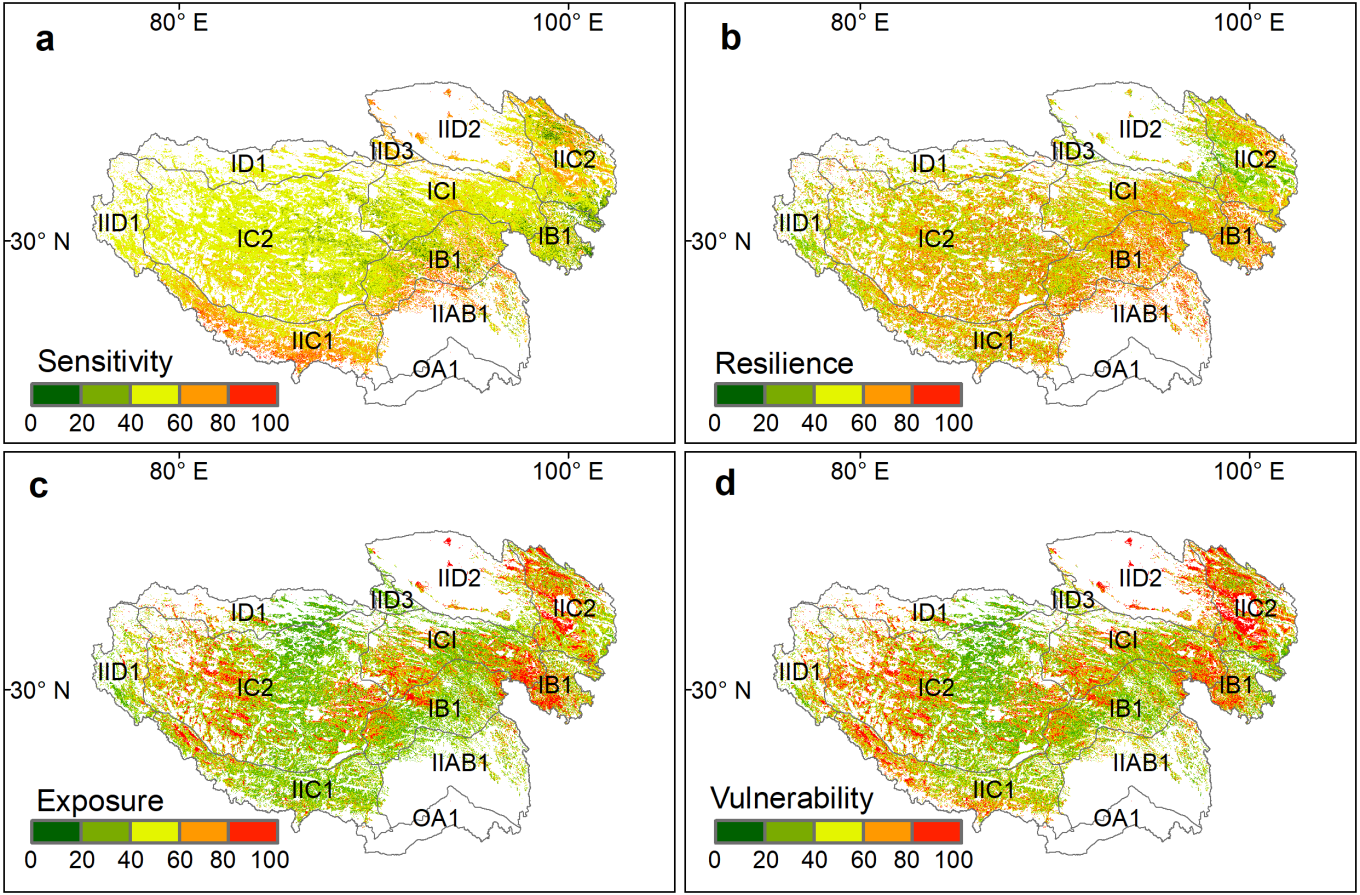
Spatial patterns of standardized grassland (A) sensitivity index (SI), (B) resilience index (RI), (C) exposure index (EI), and (D) vulnerability index (VI) on the Qinghai-Tibet Plateau.

The sensitivity, resilience, exposure, and vulnerability index of alpine grasslands showed distinct patterns on the Qinghai-Tibetan Plateau (Fig. 2). In terms of the sensitivity index, most of the alpine grasslands were moderately sensitive to climate change (40 < SI < 60), which accounted for 67.43% of grassland pixels (Fig. 2A). The extreme sensitivity level (SI > 80), accounting for only 2.72% of grassland pixels, was mainly distributed in the south of the plateau. The areas of slight and low sensitivity level were primarily located in the east of Qinghai province (SI < 20), accounting for 1.45% of the grasslands on the plateau (Table 1). For the resilience index (Fig. 2B), grasslands with slight resilience (RI < 20) accounted for 3.63% of grasslands on the whole plateau, and were mainly situated in the north of the plateau. Moderate (40 < RI < 60) and high resilience index (60 < RI < 80) areas were scattered across the whole plateau, accounting for 39.92% and 29.29% of the total grassland area, respectively.

**Table 1:** Pixel percentage (%) of the standardized sensitivity index (SI), resilience index (RI), exposure index (EI), and vulnerability index (VI) on the Qinghai-Tibet Plateau.

Index	Pixel percentage (%)				
	Slight (0–20)	Low (20–40)	Moderate (40–60)	High (60–80)	Extreme (80–100)
SI	1.45	9.04	67.43	19.37	2.72
RI	3.63	18.94	39.92	29.29	8.21
EI	20.20	28.77	25.64	12.92	12.48
VI	16.82	24.51	25.92	17.05	15.71

The spatial pattern and the proportion among five different levels of the exposure index were fairly consistent with the vulnerability index (Figs. 2C and 2D, Table 1). Highly vulnerable grasslands (60 < VI < 80) and extremely vulnerable grasslands (80 < VI < 100) together accounted for 32.76% of the total grassland areas, which were mainly distributed in the eastern and western parts of this plateau. The grasslands with a slight (VI < 20) and low vulnerability index (20 < VI < 40) accounted for 16.82% and 24.51% of the entire grassland area on the plateau, respectively, and were mainly distributed in the center of the plateau.

### Grassland vulnerability of different eco-geographical regions and grassland types

**Figure 3:**
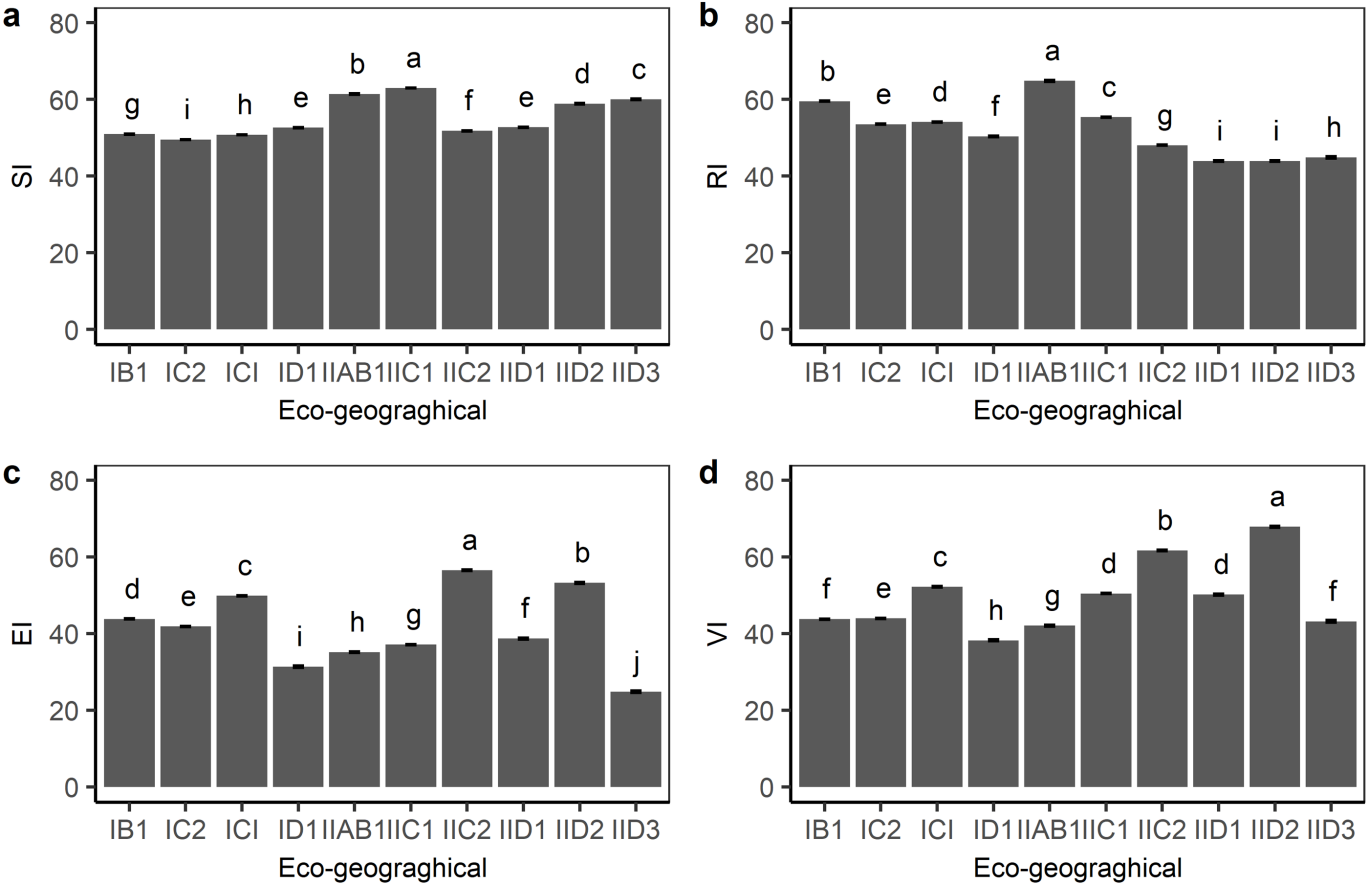
Grassland standardized (A) sensitivity index (SI), (B) resilience index (RI), (C) exposure index (EI) and (D) vulnerability index (VI) for each eco-geographical region on the Qinghai-Tibetan Plateau. Bars in the figure represent standard error.

The division of eco-geographical regions took both the characteristics of climatic factors and vegetation cover information into consideration at the same time. Except for the OA1 region, the remaining ten eco-geographic regions showed an alpine grassland distribution. The sensitivity, resilience, exposure, and vulnerability index within the ten eco-graphical regions were compared. As shown in Fig. 3, IIAB1 and IIC1 in the humid area of the eastern Qinghai-Tibetan plateau had the highest sensitivity index, whereas IC1 and IC2 in the center of the plateau had a relatively low sensitivity index. The resilience index in IIAB1 and IIC1 was higher than that in other eco-geographical regions. The vulnerability index among the ten eco-geographical regions was similar with the exposure index, but with some differences. For example, although the lowest exposure index was found in IID3, the vulnerability index in IID3 was not the lowest because of the higher sensitivity index. This indicates that the degree of exposure determined the vulnerability of alpine grasslands on the plateau, but it was also affected by grassland sensitivity and resilience simultaneously.

**Figure 4:**
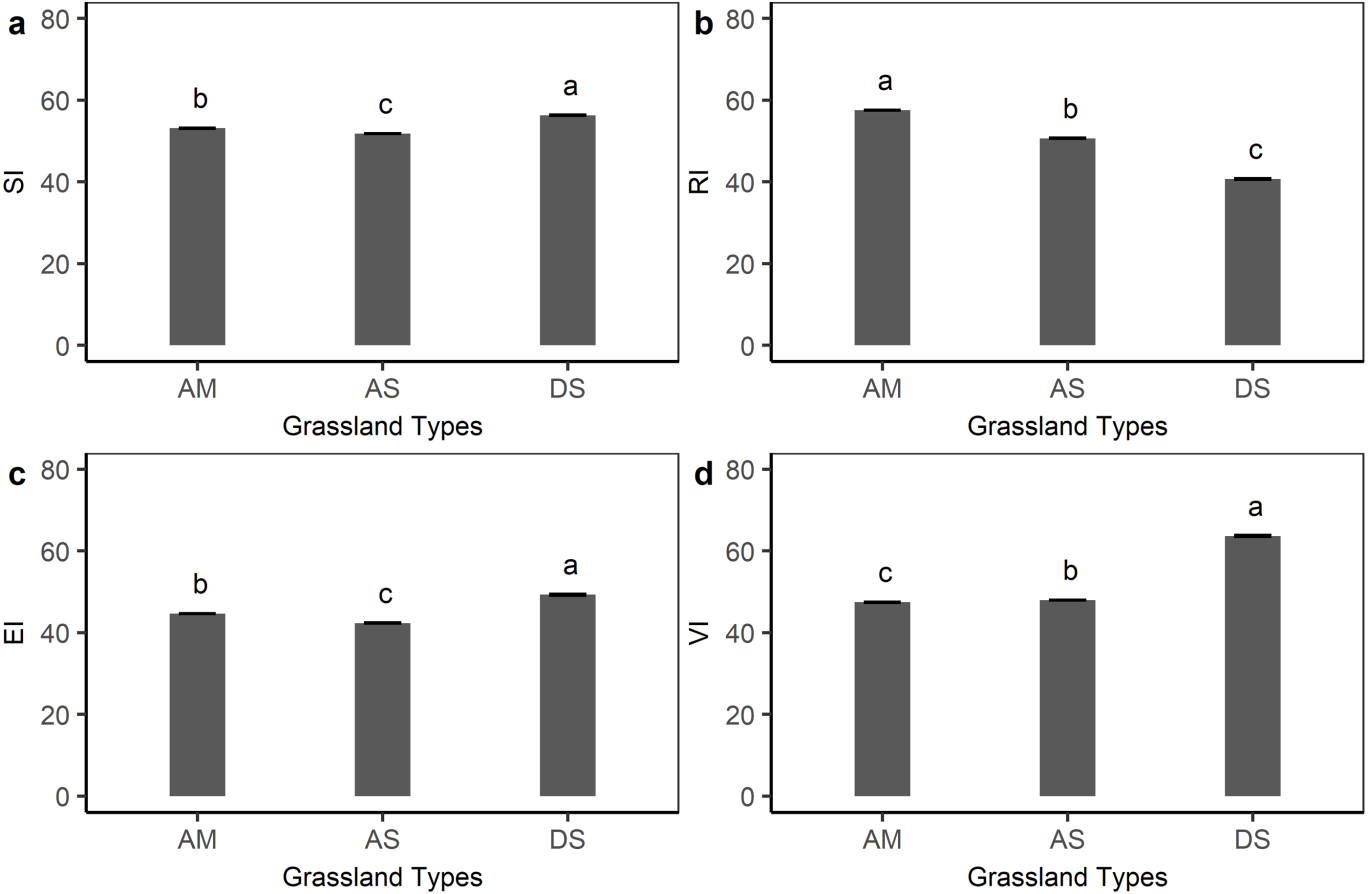
Grassland sensitivity index (SI), resilience index (RI), exposure index (EI), and vulnerability index (VI) for each grassland type on the Qinghai-Tibetan Plateau. AM, alpine meadow; AS, alpine steppe; DS, desert steppe. Bars in the figure represent standard error.

Figure 4 shows the sensitivity, resilience, exposure, and vulnerability index for different grassland types on the Qinghai-Tibetan plateau. Compared with the other three indexes, the sensitivity index was less variable among alpine meadow (53.1), alpine steppe (51.8), and desert steppe (56.3), but with a significant difference (*P* < 0.05, ANOVA test). The average resilience index of the alpine meadow (57.7) was relatively higher than desert steppe (40.7) and alpine steppe (50.6). For the exposure index, desert steppe (49.3) was significantly higher than that in the alpine steppe (42.4) and alpine meadow (44.7). Desert steppe also had the highest vulnerability to climate change (63.7), whereas alpine steppe (47.9) and alpine meadow (47.4) were subject to less vulnerability.

### The effects of grazing intensity on grassland vulnerability

**Figure 5:**
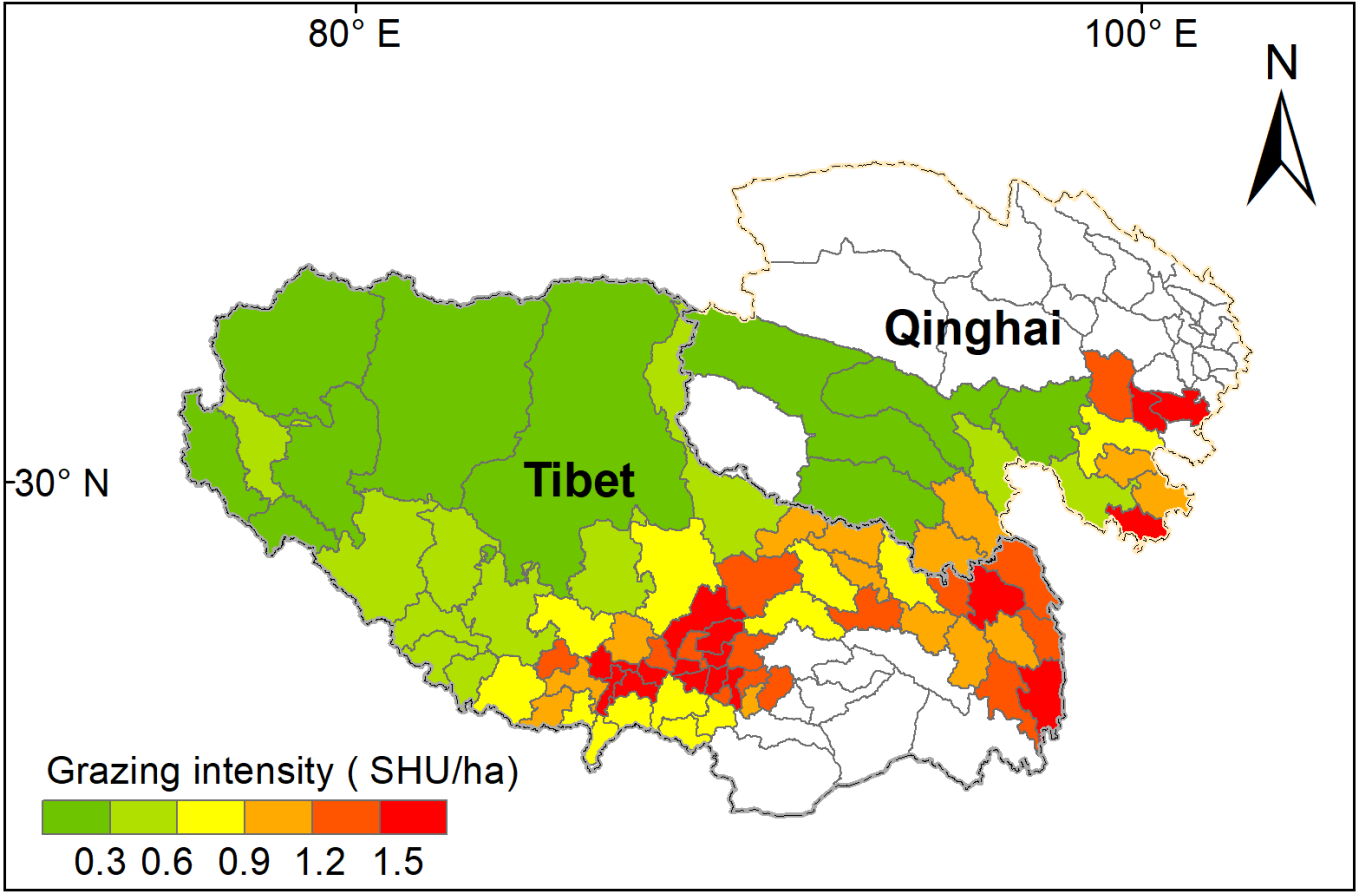
Mean grazing intensity (GI) from 2000 to 2017 for the 78 counties on the Qinghai-Tibet Plateau. White color counties in Tibetan indicate grassland areas less than 50 km^2^, and white color counties in Qinghai indicates null-value due to unavailable data. SHU represent sheep unit.

**Figure 6:**
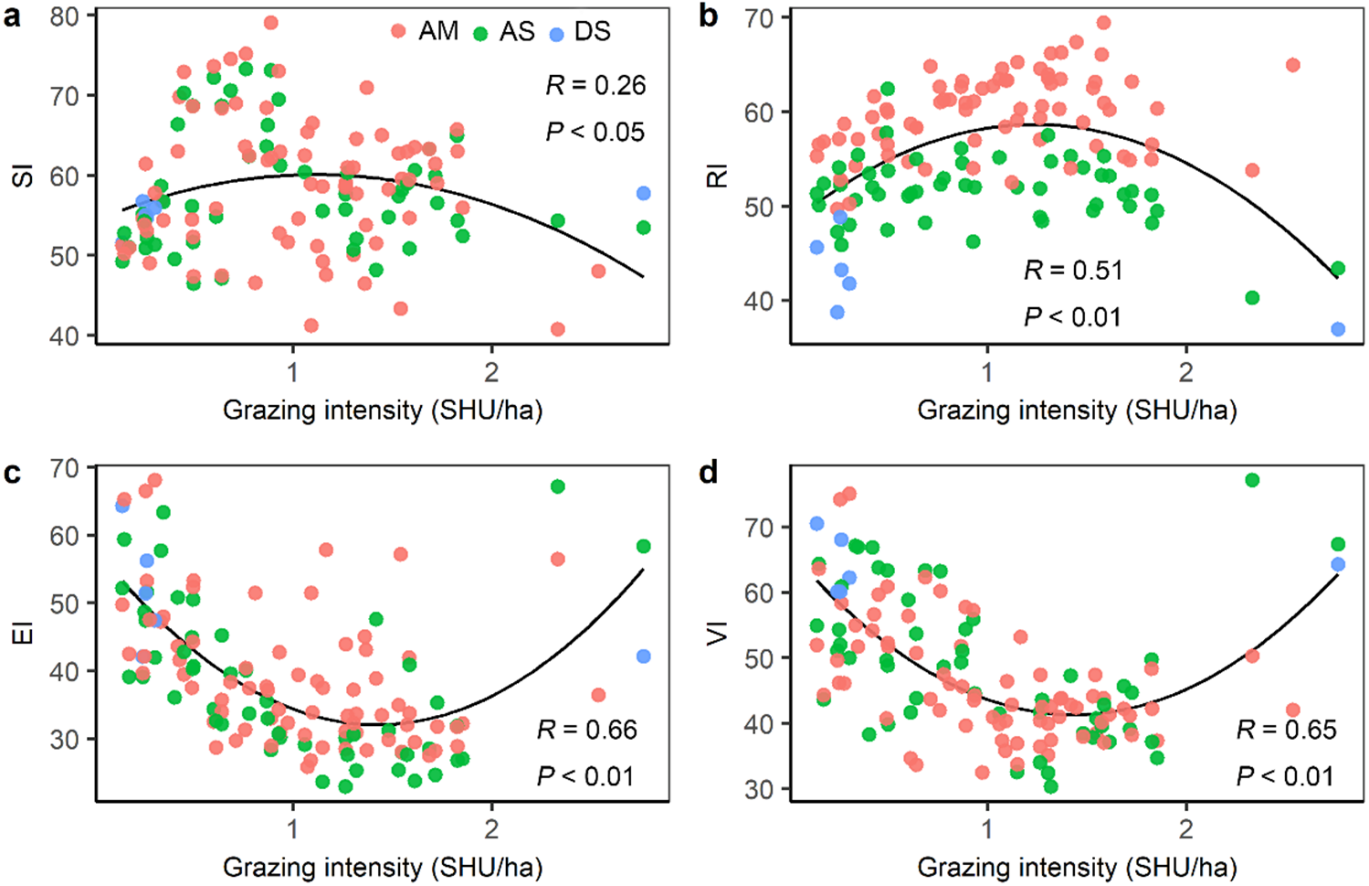
The relationship between grazing intensity and (A) sensitivity index (SI), (B) resilience index (RI), (C) exposure index (EI), and (D) vulnerability index (VI). AM, alpine meadow; AS, alpine steppe; DS, desert steppe.

Grazing intensity showed a distinct spatial pattern among counties on the Qinghai-Tibetan Plateau. Eastern and central counties in Tibet and Qinghai province had higher grazing intensity (above 1.5 SHU/ha) than western counties (below 0.3 SHU/ha) (Fig. 5). Quantitative analysis was conducted on the grazing intensity and the corresponding sensitivity index, resilience index, exposure index, and vulnerability index. As shown in Fig. 6, the index of grazing intensity had a significant impact on the vulnerability of alpine grasslands as well as the three components (*P* < 0.05). Specially, we found unimodal responses of sensitivity index and resilience index to grazing intensity across the counties (Figs. 6A and 6B). This indicates that grasslands with moderate grazing intensity might result in high sensitivity and resilience. The response of exposure index to the changes in grazing intensity was similar to that of the vulnerability index, which shown as a significant U-shaped pattern in Figs. 6C and 6D. Thus, moderate grazing intensity might play a crucial role in preventing alpine grasslands from becoming vulnerable.

### The relative contribution of grassland type and grazing intensity to the spatial pattern of vulnerability

In the GLM analysis, grazing intensity, grassland type, and the interactions between them could explain 1.46%, 64.20%, 19.42%, and 28.24% of the total variation in sensitivity index, resilience index, exposure index, and vulnerability index, respectively (Table 2). For grassland vulnerability, we found that grassland type and grazing intensity accounted for 10.34% and 15.36% of its spatial variation, respectively. This indicates that grazing intensity had a relatively higher explanatory power than grassland type for the spatial variation in the vulnerability index.

**Table 2:** Summary of the effects of grazing intensity (GI), grassland types (TYPE), and the interactions between them in general linear models (GLMs) on the sensitivity, resilience, exposure, and vulnerability indices on the Qinghai-Tibet Plateau.

Index	Explicators	d.f.	SS	MS	F	P	%SS
SI	GI	1	7.80	7.84	0.13	0.72	0.10
	Type	2	77.70	38.87	0.62	0.54	0.98
	GI:Type	2	30.60	15.32	0.25	0.78	0.38
RI	GI	1	55.34	55.34	3.77	0.05	1.07
	Type	2	2993.90	1496.95	102.08	0.00	58.01
	GI:Type	2	264.04	132.02	9.00	0.00	5.12
							
EI	GI	1	2211.40	2211.44	23.42	0.00	14.98
	Type	2	590.20	295.12	3.13	0.05	4.00
	GI:Type	2	65.00	32.51	0.34	0.71	0.44
							
VI	GI	1	2165.50	2165.45	26.97	0.00	15.36
	Type	2	1457.50	728.75	9.08	0.00	10.34
	GI:Type	2	357.70	178.86	2.23	0.11	2.54

**Notes.**

d.f. degree of freedom SS sum squares MS mean squares F variance ratio P significance %SS percentage of the total sum of squares explained

## Discussion

With the increased variability of climate change, it is essential to understand the vulnerability of ecosystems, especially for high-altitude vegetation which is believed to be highly sensitive to climate change. Sensitivity, resilience, and exposure are essential components of vulnerability reflecting the degree of an ecosystem exposure and response to perturbations (Williams et al., 2008). In this study, the vulnerability of alpine grasslands on the Qinghai-Tibetan Plateau was calculated based on the combination of external (exposure) and intrinsic (sensitivity and resilience) components. In the following sections, we will discuss the spatial pattern of vulnerability and the driving factors for this pattern in detail.

### The spatial pattern of grassland vulnerability

We found that grasslands with a high vulnerability were mainly located on the edges of the plateau, while grasslands with a low vulnerability were distributed in the hinterlands of the plateau. The spatial pattern of vulnerability resembles the exposure pattern. The results indicate that the exposure index was the crucial component in determining the vulnerability of the grassland ecosystems on the Qinghai-Tibetan Plateau, which is partially consistent with previous studies. For example, Li et al. (2018) found that the vulnerability of ecosystems is mainly determined by the degree of exposure. Beaugrand et al. (2015) also suggested that the influences of exposure to biodiversity were stronger than those of sensitivity. Exposure is an indicator reflecting the ability of species to keep pace with the changing climates (Loarie et al., 2009), which highly depends on the degree of regional climate change that involves the range of species and habitats. Species need a survival environment to be within a particular range for vital growth and reproduction processes (Burrows et al., 2011). Once environmental variability exceeds the tipping point that species unable to cope with, the shift of biogeographic ranges may occur and the ecosystem may become increasingly vulnerable (Beisner, Haydon & Cuddington, 2003). Thus, environmental variability is particularly important for species richness, community structure, and biodiversity (Chen et al., 2011; Lindner et al., 2010), and thereby can determine the degree of ecosystem vulnerability. However, some differences between the spatial pattern of exposure and the spatial pattern of vulnerability also occurred in some sub-regions. For example, alpine grasslands in the southern Qinghai-Tibetan Plateau were exposed to low climate variation but with high vulnerability. This may be because the differences in resilience and sensitivity might result in various responses of an ecosystem to the same climate change. This result provides evidence to support the framework that ecosystem vulnerability assessment should integrate sensitivity and resilience simultaneously.

As illustrated above, the effects of climate change on ecosystems were not only determined by the magnitude and distribution of perturbations but also influenced by the ability of the target ecosystem to resist perturbations. High ecological sensitivity is likely to exacerbate ecosystem vulnerability, whereas high ecological resilience is likely to alleviate ecosystem vulnerability. In this study, we found that vegetation dynamics in response to climate change varied among grassland types. Earlier studies also have revealed that the sensitivity of grasslands on the Qinghai-Tibetan Plateau to the changing climate change is complex and often varies dramatically among regions and grassland types (Li et al., 2019a). For example, grasslands on the southeastern Qinghai-Tibetan Plateau are generally sensitive to temperature variations while northeastern grasslands show strong responses to precipitation variations (Huang et al., 2016; Sun, Qin & Yang, 2016). With regard to the differences in ecological sensitivity among three grassland types, we found that desert steppes were more sensitive to climate change than alpine meadows and alpine steppes. Our result is in line with previous studies showing that grasslands in drier and warmer regions are highly sensitive to precipitation (Li et al., 2019a). Li et al. (2019b) also suggested that desert steppes are likely to have high sensitivity to the timing variability of precipitation on the Northern Tibetan Plateau. One potential explanation is that desert steppes are always characterized by poor species and low vegetation productivity due to low precipitation (Wu, Shen & Zhang, 2014), but with high precipitation variation which can result in rapid changes in the key carbon cycle process (Knapp et al., 2002). The resilience metric in our study is a kind of engineering resilience, emphasizing the maintenance of ecosystem function effectiveness rather than the probability of an ecosystem switching to another state (De Keersmaecker et al., 2015). This kind of resilience is closely linked with ecosystem function and plant community composition (Hoover, Knapp & Smith, 2014). Xu et al. (2016) indicated that high productivity and adaptive dominant species might result in high resilience. Therefore, alpine meadows with higher productivity and larger species richness pool might have a higher ability to recover from perturbations than alpine steppes and desert steppes (Wu, Shen & Zhang, 2014; Zhu, Jiang & Zhang, 2016). Besides, we also found that desert steppes exhibited high sensitivity and low resilience, indicating that the variations in grassland biomass for desert steppes are higher than alpine meadows and alpine steppes if the ecosystems experiencing same extrinsic perturbations.

### Grassland type and grazing intensity affect grassland vulnerability

In the GLM, we found that both grassland types and grazing intensity were significant in driving the spatial variation of grassland vulnerability. First, the vulnerability differed dramatically among grassland types. Desert steppes were significantly more vulnerable than alpine meadows and alpine steppes. The high vulnerability of desert steppes might result from high sensitivity, high exposure, and low resilience. For the three grassland types on the Qinghai-Tibetan Plateau, we found that high exposure was always connected with high sensitivity. This finding is consistent with the previous study, which suggests that ecosystems with high perturbations are more sensitive to climate change than ecosystems with low perturbations over short timescales (Kröel-Dulay et al., 2015). Moreover, in this study, human activities were not considered when calculating the vulnerability index; however, the alpine grassland’s vulnerability was significantly correlated with grazing intensity which is considered as the primary human activity. This finding indicates that anthropogenic disturbances to ecosystems should not be ignored. Grazing is the main form of land use for most grassland ecosystems, which also affects the vulnerability of ecosystems to climate change (Christensen et al., 2004; Izaurralde et al., 2011). Multiple experiments have demonstrated that grazing affects the stability and self-regulating ability of the ecosystem by changing grasslands’ structure and function, such as grassland productivity, biodiversity, plant community composition and water-use efficiency (Wang et al., 2012; Zhang et al., 2015a). These ecosystem changes could result in changes in the sensitivity (Christensen et al., 2004) and the resilience (Li, 1997) of vegetation to climate change or even the degree of exposure. In our study, a significant U-shaped relation was found between grazing intensity and vulnerability index, indicating that moderate grazing intensity might play a crucial role in mitigating the grassland vulnerability. This result is in line with the intermediate disturbance hypothesis, which suggests that human influences in the moderate or middle range can promote community succession and maintain community structure and species diversity (Grime, 1973), and thereby prevent ecosystems becoming vulnerable. The interactions of climate change and livestock grazing play a significant role in shaping grassland functions (Watson et al., 2000). In the future, more frequent and intense climate changes and more pressures on grasslands for their resources are expected (Kharin et al., 2013; Venter et al., 2016). Thus, it is necessary to investigate interactions between climate change and grazing, to prevent grasslands from being vulnerable and to maintain sustainable grazing systems.

### Implications for managers and policymakers

More information about climate and human activities changes should be supplied to policymakers and herdsmen, to develop region-specific policies and sustainable management strategies. For the vast grassland on the Qinghai-Tibetan Plateau, it is necessary to use simple ecological indicators that reflect the information about the status and health of ecosystems to identify the priority areas. We first recommend that policymakers should pay more attention to western desert steppes because of the high vulnerability resulting from high exposure, high sensitivity, and low resilience. Once experience strong climatic perturbations, grasslands in these regions are easy to collapse, and do not easily recover. Optimizing allocation of management, such as monitoring ecological processes and functioning of grassland ecosystems and curbing overgrazing, is needed to maximize the grassland resistance and resilience to perturbations. Second, we highlight the necessity to conduct studies to predict future vulnerability. Predicting how ecosystems respond to climate change is useful to make adaptation and mitigation strategies to alleviate the effects of climate change on ecosystems.

## Conclusions

Climate change is affecting the vulnerability of alpine grasslands on the Qinghai-Tibetan Plateau. In this study, we quantified the vulnerability of grasslands with the combination of the sensitivity, resilience, and exposure indices according to the definition by the IPCC. The vulnerable grasslands were mainly distributed on the sides of the Qinghai-Tibetan Plateau. Exposure was the dominant index for this vulnerability pattern; however, the sensitivity and resilience of alpine grasslands could also exacerbate or alleviate the degree of vulnerability. Although human activities were not considered in calculating vulnerability, the grazing intensity had a significant impact on the spatial pattern of grassland vulnerability. We therefore suggest that anthropogenic factors should be considered in the assessment of ecosystem vulnerability in the future.

##  Supplemental Information

10.7717/peerj.8513/supp-1 Supplemental Information 1 The sensitivity index (SI), resilience index (RI), exposure index, and (d) vulnerability index (VI) under different grazing intensity

## Additional Information and Declarations

### Data Availability

The following information was supplied regarding data availability: The raw measurements are available in a Supplemental File.
